# Genetic Investigation of Beta-Lactam Associated Antibiotic Resistance Among *Escherichia Coli* Strains Isolated from Water Sources

**DOI:** 10.2174/1874285801711010203

**Published:** 2017-09-22

**Authors:** Reza Ranjbar, Mehrdad Sami

**Affiliations:** 1 Molecular Biology Research Center, Baqiyatallah University of Medical Sciences, Tehran, Iran; 2 Department of Clinical Sciences, School of Veterinary Medicine, Ferdowsi University of Mashhad, Mashhad, Iran

**Keywords:** Antibiotic, Bacterial resistance, *Escherichia coli*, ESBL, PCR, Water sources

## Abstract

**Background:**

Antimicrobial resistance is an important factor threatening human health. It is widely accepted that antibiotic resistant bacteria such as *Escherichia coli* (*E. coli)* released from humans and animals into the water sources, can introduce their resistance genes into the natural bacterial community.

**Objective:**

The aim of this study was to investigate the prevalence of *bla_TEM_, bla_CTX_, bla_SHV_, bla_OXA_* and *bla_VEB_* associated-antibiotic resistance among *E. coli* bacteria isolated from different water resources in Iran.

**Methods:**

The study contained all *E. coli* strains segregated from different surface water sources. The Kirby-Bauer method and combined discs method was determined in this study for testing antimicrobial susceptibility and strains that produced Extended-Spectrum Beta Lactamases (ESBL), respectively. DNA extraction kit was applied for genomic and plasmid DNA derivation. Finally the frequency of resistant genes including *bla_TEM_, bla_CTX_, bla_SHV_, bla_OXA_* and *bla_VEB_* in ESBL producing isolates were studied by PCR.

**Results:**

One hundred *E. coli* strains were isolated and entered in the study. The highest antibiotic resistance was observed on clindamycin (96%). Moreover, 38.5% isolates were ESBL producers. The frequency of different ESBLs genes were 37%, 27%, 27%, and 25% for *bla_TEM_, bla_CTX_, bla_SHV_, and bla_OXA_*, respectively. The *bla_VEB_* wasn’t found in any isolates.

**Conclusion:**

The study revealed a high prevalence of *CTX-M, TEM, SHV* and *OXA* genes among *E. coli* strains in surface water resources. In conclusion, these results raised a concern regarding the presence and distribution of these threatening factors in surface water sources and its subsequent outcomes.

## 1 INTRODUCTION

The increase in number of resistant strains of bacteria and transmission of resistance genes from environmental bacteria to human pathogenesis are an important factor threatening human public health, so that become a major concern health authorities worldwide [1, 2]. Extended-spectrum beta-lactamases (ESBL) is responsible for creating resistant strains of bacteria against antibiotics [3-5]. The spread of ESBL-producing strains limited to antimicrobial agents for the treatment of patients effectively and there is a concern on *Escherichia coli (E. coli)*, and most of other pathogen such as, *Shigella*, *Salmonella*, and *Klebsiella* [6-9]. *E. coli* associated diseases and water-borne outbreaks result in high morbidity and mortality worldwide [10]. Bacterial contamination of surface water resources has long been considered as a water quality issue which can be contribute in disease transmission. Bacteria resistant to antibiotics are one of the most dangerous biological contaminants in surface water sources [1]. Hydrolysis of β-lactam ring structure by ESBL caused antibiotic function impairment. ESBL enzymes are able to target a broad spectrum of antibiotics, such as penicillins and their complexes, monobactams, and new generation cephalosporins [11]. There are hundreds types of β-lactamase enzymes, each ESBL bacterium may possess genes for one or more of these enzymes. Similar *bla* genes with alike structure are grouped together within these gene families in terms of phylogenetic and by apply PCR are often targeted for detection and identification. *TEM* and *OXA* as a kind of ESBL, contain resistance genes against β-lactamase inhibitors, creating such species more of a threat [12]. Transfer of resistance genes between bacteria is easily facilitated because the genes are encoded on mobile vectors (plasmids and transposons) [13, 14].

The use and misuse of antimicrobial drugs in hospitals can lead to development of antibiotic resistant bacteria and the spread of resistant organisms such as *E. coli* in the environment including swage and surface waters. *E.coli* is a potential uropathogenic bacterium which may cause a wide range of Urinary Tract Infections (UTIs) including asymptomatic and/or symptomatic bacteriuria, cystitis, and pyelonephritis both in children and adults. Moreover, detection of this organism in water is an implicit indicator of fresh fecal pollution and consequently of the hazard of co-occurrence of enteric pathogens that can reason illness in susceptible populations. Access to adequate amounts of safe and healthy foods is key to supporting life and promoting good health of people [15, 16]. A high incidence of Multiple Antibiotic Resistances (MAR) has been detected in *E. coli* samples isolated from river and contaminated food or water. MAR in intestinal bacteria is originating from wastewater treatment plants [17-20]. This problem is clinically important in patients with problems associated with *E. coli*, for example: uropathogenic *E. coli* strains had the highest resistance to penicillin (100%) and ampicillin (80.20%) while the lowest resistance rate was observed to imipenem (0%) and nitrofurantoin (5.69%) [21-23].

Analysis of phenotypic susceptibility to penicillin and cephalosporins can obtain an accurate assessment of β-lactamase producing *Enterobacteriaceae* [24]. β-lactamases are modifying enzymes (drug-inactivating enzymes) found in all types of bacteria. The efficiency of β-lactam antibiotics in eradicating infection at various body sites can diminish by β-lactamase-producing organisms [25].

By use of DNA-based technologies, a new era seems to be opening in the field of diagnostic and molecular epidemiology of antibiotic resistant bacteria. The application of PCR based techniques has had a revolutionary impact in rapid detection of resistance determinants, such as ESBLs genes [26, 27].

This study was conducted for evaluating the prevalence of *bla_TEM_, bla_CTX_, bla_SHV_, bla_OXA_* and *bla_VEB_* associated-antibiotic resistance among *E. coli* strains which are isolated from different water resources in Iran.

## 2 MATERIALS AND METHODS

A total of 100 water samples which have been taken earlier from different water sources during one year between September 2012 and September 2013 was included for the study [28].Water samples were collected in the sterilized bottles with a capacity of 200 ml. The multiple tube fermentation method was considered for sampling of the coliform group components and presence–absence coliform test. The water samples were chilled on ice packs immediately after collection to prevent any changes in the microbial flora of the samples during transportation to laboratory. Samples were analyzed within 2 h of collection. In order to hold *E. coli* isolates until the end of collecting samples process, all isolates in culture media of brain-heart infusion (BHI) contained 25% glycerol and were maintained at a temperature of -70°C.

Each water samples were inoculated in Lauryl sulfate broth and PA broth, respectively. All the inoculated tubes were incubated at 35±0.5ºC for 24-48hrs. Following turbidity and gas in the durham tube and create an acidic pH in the samples, the presence of coliform was primarily approved and given to the next-step verification for further consideration. All samples showing production of gas, acid and turbidity were cultured in brilliant green bile broth and incubated at 35±0.5ºC for 24-48hrs. The presence of coliforms in samples confirmed by gas production and acid pH. In the next step of diagnosis process, all samples showing production of gas, acid and turbidity were cultured in EC broth and incubated at 44.5±0.2ºC for 24 hrs. The presence of coliforms in samples that tolerated to heat confirmed by gas production and acid pH. In the next step, the samples that were positive in this phase were cultured on selective culture media of eosin methylene blue agar and incubated at 35±0.5ºC for 24-48 h. The presence of *E. coli* was confirmed by observation of colonies with green metallic sheen. All colonies containing metallic sheen were tested by IMViC test and if the results were compatible, they would have been isolated and storaged as strains of *E. coli*.

The dilution and disk-diffusion methods were applied in this study for evaluation of antimicrobial susceptibilities of the *E. coli* bacteria. Antibiotic discs were placed within a distance of 12 mm from each other and incubated at 37ºC for 18 hrs. To determine the production of ESBL enzymes in accordance with the instructions of CLSI, the screening method of combined discs was used. Antibiotic discs were placed within a distance of 15 mm from each other and incubated at 37ºC for 24 hrs. Antibiotics, including clindamycin, lincomycin, rifampin, ceftazidime, streptomycin, cefazolin, tetracycline, gentamicin, nalidixic acid, kanamycin, trimethoprim, tobramycin, ciprofloxacin, cloramfenicol, imipenem, norfloxacin, amikacin, azithromycin, and nitrofurantoin were tested in this study. The phenotypic confirmatory test was applied for identification of ESBL producers by using cefotaxime and ceftazidime individually and also combined with clavulanic acid [29].

A kit that extract AccuPrep® genomic DNA (Bioneer, South Korea) was applied for extraction of DNA. Absorbance of the samples at 260 nm examined the DNA concentration [30]. The five specific primers [31, 32] were selected to expanding genes under this study and for each one a forward primer and a reverse primer was chosen (Table **1**). PCR reaction was performed in a DNA thermocycler as follows: Primary denaturation at 96ºC for 5 min, denaturation by 30 cycles at 95ºC as long as 1 min, annealing at 55ºC within 1 min (based on the type of primer for each gene is different from that given in Table **1**), elongation at 72ºC as long as 1 min and extension phase at 72ºC for 7 min, completed by a hold at 4ºC. PCR products were electrophoresed on 1.5% agarose gel containing ethidium bromide at 80 V for 1 h.

## 3 RESULTS

Totally, 100 *E. coli* isolates were isolated. In the present study, the antibiotic resistance rates to different antibiotics is given in Fig. (**1**). The highest and lowest rates of antibiotic resistance were related to clindamycin (96%) and nitrofurantoin (1%), respectively.

The frequency of resistance genes were 37, 27, 27, and 25 into different strains based on the detection of the various resistance genes including *bla_TEM_, bla_CTX_, bla_SHV_, and bla_OXA_*, respectively. The *bla_VEB_* gene was not distinguished at all items. Also, at the same time, 37% of the samples showed resistance to more than one gene (11, 12, and 14 percent of isolates showed resistance genes to two, three and four ones).

## 4 DISCUSSION

*E. coli* bacteria through water plays a role in the transmission of different types of diseases. Presence of this bacteria in the water, proceeds to the release of these bacteria in the environment which involved in antibiotic resistance. This resistance can be due to the presence of specific genes of ESBL such as, *bla_TEM_, bla_CTX_, bla_SHV_, bla_OXA_ and bla_VEB_*. Knowing the types and frequency of these genes made a decision for the treatment process of patients effectively.

The present study investigated the genetic study of beta lactam associated antibiotic resistance among *E. coli* bacteria from water resources and reported rate of resistance to antibiotics. High level of resistance in 100 isolates of this bacteria isolated from different water resources mostly shown to clindamycin (96%), lincomycin (94%), rifampin (93.6), cefazolin (54.2) and ceftazidime (39.6). ESBL genes and the frequency of *bla_TEM_, bla_CTX_, bla_SHV_ and bla_OXA_* genes were apprehended, while Kim *et al.* [33] detected sixteen strains of *E. coli* that were highly resistant to about 10 antibiotics. Resistance to cephalothin, ampicillin, tetracycline, streptomycin, sulfixosazole, trimethoprim, kanamycin, and gentamicin have been shown in all ESBL-producing *E. coli* strains. Further, Kim *et al.* [33] only reported *bla_TEM_* and *bla_CTX_* as ESBL. Jang *et al.* showed that with the dominance of the bla_CTX_, beta-lactamase gene had more than one gene in 67% of cases [34]. In another study that was conducted on the Thames river water samples, the greatest resistance mainly caused by *bla_CTX-M14_* gene for *E. coli* that used ciprofloxacin and ampicillin [35].

Toroglu *et al.* [36] isolated 67 strains from Aksu river which 67,2% were *E. coli* organism. Multiple antibiotic resistances were determined in 27 isolates (40%) in which they were resistant to five or more antibiotics and 49,3% of the isolates were found to be Beta-lactamase producing bacteria. A high antibiotic resistance of *E. coli* to antibiotics was indicated in this study.

ESBLs and carbapenemases considered as important mechanisms in *Enterobacteriaceae* that cause modern broaded-spectrum cephalosporin and carbapenem resistance. A great concern is the extension of ESBL and carbapenemase producers into the environment. ESBL-producing *Enterobacteriaceae* were detected in 21 (36.2%) of samples. The expression of an ESBL phenotype was e shown (74 strains confirmed). *SHV-12* ESBL producers were identified in three different strains, the genes encoding *CTX-M* ESBLs were presented in 71 strains [37].

The presence of circumstance of *CTX-M* (84.2%) and *TEM* (47.4%) genes in isolates from urban surface waters in Malaysian were mostly *E. coli* and *K.* *pneumoniae* [38].

Change of antibiotic resistance following the change in water quality is leading to negative effects on Public health. High frequency of resistant bacteria against amoxicillin and ampicillin has been reported by Muleta T. *et al.* [39]. Haque *et al.* examined surface water in Dhaka, revealing *E. coli* as the most prevalent isolated among *Enterobacteriaceae*. The spread of multidrug resistant bacteria may be affected by *CTX-M* and *SHV* types ESBL genes [40].

From the 767 *E. coli* isolates in wastewater treatment plants, the highest resistance rates were found for ampicillin and piperacillin; cefalothin and cefuroxime-axetil; nalidixic acid; trimethoprim/sulfamethoxazole and tetracycline [41].

Chatterjee *et al.* revealed that susceptibility rate was high (*i.e.* 85%) in case of Cefixime. It was 75% and 30% against Streptomycin and Ciprofloxacin among the twenty *E.coli* isolates, respectively. High level of resistance was reported over Doxycycline, since all isolates were found to be resistant against this antibiotic. Cefixime was detected as the most impressive antimicrobials against *E. coli* isolates [42].

In addition, the results of 27,886 isolates of *E. coli* from Minjiang river in China showed most resistant to cefoperazone, cefazolin, ciprofloxacin, norfloxacin and rifampicin [43].

To the best of our knowledge, this is the first study on evaluation of the prevalence of *bla_TEM_, bla_CTX_, bla_SHV_, bla_OXA_ and bla_VEB_* associated antibiotic-resistance among *E. coli* bacteria isolated from different water resources in Alborz province of Iran. This study revealed high levels of antibiotic resistance and prevalence of *bla_TEM_, bla_CTX_, bla_SHV_ and bla_OXA_* genes that were detected in *E. coli* isolations from water samples. Increasingly, the risk of antibiotic resistance gene transfer rises among bacteria in the water sources. This is a serious warning, because ESBLs are an eminent threat to the efficacy of antibiotics that are available in current medical uses.

*E. coli* producer isolates have now risen to prominence, mainly associated with community-acquired infections. Iran, like many other regions of the world, has experienced a significant increase in the numbers of ESBL-producing bacteria in the new millennium. The high prevalence of ESBL genes in Iran means that the empirical treatment of serious infections with beta-lactam antibiotics is seriously compromised. In addition to human and animal health risks from infectious microorganisms, potential hazards associated with the application of sewage sludge to agricultural land include contamination of surface and ground waters. In this paper, we presented evidence of a greater frequency of fecal coliforms and drug-resistant bacteria in surface waters.

Our results revealed a high prevalence of ESBLs genes among *E. coli* strains in surface water resources. The antimicrobial drug resistance that is increasingly compromising the usefulness of the b-lactam antibiotics and other previously life-saving antibacterial drugs.

## CONCLUSION

In conclusion, these results raised a concern regarding the presence and distribution of these threatening factors in the surface water sources and its subsequent outcomes. Prevention and control strategies should be urgently implemented to stop further spreading of these strains.

## Figures and Tables

**Fig. (1):**
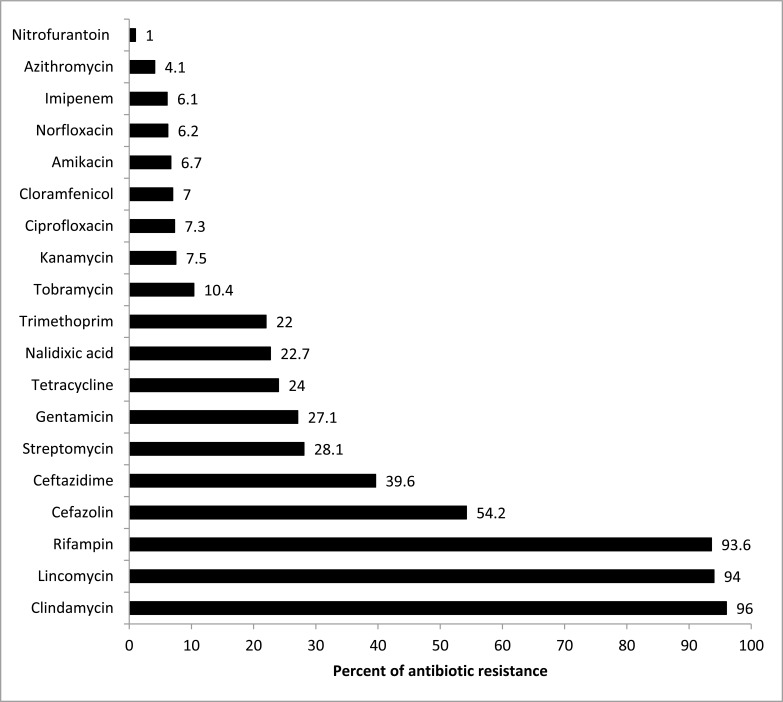
Antibiotic resistance against *E. coli* isolated from river water in Alborz province, Iran.

**Table 1:** Primers used to amplify the genes of beta-lactamase.

Target Type of β-Lactamase Genes	**Primer Sequence**	**Size of** **Product (bp)**	**AT (**ºC**)**
*bla_SHV_*	5´-TTA ACT CCC TGT TAG CCA-3´ (F)	860	51.2
	5´-GAT TTG CTG ATT TCG CCC-3´ (R)		
*bla_TEM_*	5´-ATG AGT ATT CAA CAT TTC CG-3´ (F)	867	52.2
	5´-CTG ACA GTT ACC AAT GCT TA-3´ (R)		
*bla_OXA_*	5´-ACA CAA TAC ATA TCA ACT TCG -3´ (F)	885	60
	5´-AGT GTG TTT AGA ATG GTG ATC-3´ (R)		
*bla_VEB_*	5´-CGA CTT CCA TTT CCC GAT GC-3´ (F)	1014	55
	5´-GGA CTC TGC AAC AAA TAC GC-3´ (R)		
*bla_CTX_*	5´-TTT GCG ATG TGC AGT ACC AGT AA- 3´ (F)	560	60
	5´-CTC CGC TGC CGG TTT TAT -3´ (R)		

## References

[1] Cernat R, Lazăr V, Balotescu C, Cotar A, Coipan E, Cojocaru C

[2] Walia S.K., Kaiser A., Parkash M., Chaudhry G.R.

[3] Bush K. (2008). Extended-spectrum β-lactamases in North America, 1987-2006.. Clin. Microbiol. Infect..

[4] Cantón R., Novais A., Valverde A., Machado E., Peixe L., Baquero F., Coque T.M. (2008). Prevalence and spread of extended-spectrum β-lactamase-producing *Enterobacteriaceae* in Europe.. Clin. Microbiol. Infect..

[5] Hawkey P.M. (2008). Prevalence and clonality of extended-spectrum β-lactamases in Asia.. Clin. Microbiol. Infect..

[6] Picozzi S.C., Casellato S., Rossini M., Paola G., Tejada M., Costa E., Carmignani L. (2014). Extended-spectrum beta-lactamase-positive *Escherichia coli* causing complicated upper urinary tract infection: Urologist should act in time.. Urol. Ann.

[7] Ranjbar R., Ghazi F.M., Farshad S., Giammanco G.M., Aleo A., Owlia P., Jonaidi N., Sadeghifard N., Mammina C. (2013). The occurrence of extended-spectrum β-lactamase producing *Shigella spp.* in Tehran, Iran.. Iran. J. Microbiol..

[8] Ranjbar R., Giammanco G.M., Aleo A., Plano M.R., Naghoni A., Owlia P., Mammina C. (2010). Characterization of the first extended-spectrum β-lactamase-producing nontyphoidal *Salmonella* strains isolated in Tehran, Iran.. Foodborne Pathog. Dis..

[9] Ghafourian S., Bin Sekawi Z., Sadeghifard N., Mohebi R., Kumari Neela V., Maleki A., Hematian A., Rhabar M., Raftari M., Ranjbar R. (2011). The prevalence of ESBLs producing *Klebsiella pneumoniae* isolates in some major hospitals, Iran.. Open Microbiol. J..

[10] Ranjbar R., Hosseini S., Zahraei-Salehi T., Kheiri R., Khamesipour F. (2016). Investigation on prevalence of *Escherichia coli* strains carrying virulence genes ipaH, estA, eaeA and bfpA isolated from different water sources.. Asian Pac. J. Trop. Dis..

[11] Livermore D.M. (1995). beta-Lactamases in laboratory and clinical resistance.. Clin. Microbiol. Rev..

[12] Naas T., Zerbib M., Girlich D., Nordmann P. (2003). Integration of a transposon Tn1-encoded inhibitor-resistant β-lactamase gene, bla(TEM-67) from Proteus mirabilis, into the *Escherichia coli* chromosome.. Antimicrob. Agents Chemother..

[13] Schink A.K., Kadlec K., Schwarz S. (2011). Analysis of bla(CTX-M)-carrying plasmids from *Escherichia coli* isolates collected in the BfT-GermVet study.. Appl. Environ. Microbiol..

[14] Bailey J.K., Pinyon J.L., Anantham S., Hall R.M. (2011). Distribution of the blaTEM gene and blaTEM-containing transposons in commensal *Escherichia coli*.. J. Antimicrob. Chemother..

[15] Ranjbar R., Masoudimanesh M., Dehkordi F.S., Jonaidi-Jafari N., Rahimi E. (2017). Shiga (Vero)-toxin producing *Escherichia coli* isolated from the hospital foods; virulence factors, o-serogroups and antimicrobial resistance properties.. Antimicrob. Resist. Infect. Control.

[16] Behzadi P., Najafi A., Behzadi E., Ranjbar R. (2016). Microarray long oligo probe designing for *Escherichia coli*: an in-silico DNA marker extraction.. Cent. European J. Urol..

[17] Iversen A., Kühn I., Franklin A., Möllby R. (2002). High prevalence of vancomycin-resistant *enterococci* in Swedish sewage.. Appl. Environ. Microbiol..

[18] Selvaratnam S., Kunberger J.D. (2004). Increased frequency of drug-resistant bacteria and fecal coliforms in an Indiana Creek adjacent to farmland amended with treated sludge.. Can. J. Microbiol..

[19] Jahandeh N., Ranjbar R., Behzadi P., Behzadi E. (2015). Uropathogenic *Escherichia coli* virulence genes: invaluable approaches for designing DNA microarray probes.. Cent. European J. Urol..

[20] Tajbakhsh E., Khamesipour F., Ranjbar R., Ugwu I.C. (2015). Prevalence of class 1 and 2 integrons in multi-drug resistant *Escherichia coli* isolated from aquaculture water in Chaharmahal Va Bakhtiari province, Iran.. Ann. Clin. Microbiol. Antimicrob..

[21] Farshad S., Ranjbar R., Japoni A., Hosseini M., Anvarinejad M., Mohammadzadegan R. (2012). Microbial susceptibility, virulence factors, and plasmid profiles of uropathogenic *Escherichia coli* strains isolated from children in Jahrom, Iran.. Arch. Iran Med..

[22] Momtaz H., Karimian A., Madani M., Safarpoor Dehkordi F., Ranjbar R., Sarshar M., Souod N. (2013). Uropathogenic *Escherichia coli* in Iran: serogroup distributions, virulence factors and antimicrobial resistance properties.. Ann. Clin. Microbiol. Antimicrob..

[23] Ranjbar R.E., Haghi-Ashtiani M.T., Jafari N.J., Abedini M. (2009). The prevalence and antimicrobial susceptibility of bacterial uropathogens isolated from pediatric patients.. Iran. J. Public Health.

[24] Jarlier V, Bismuth R, Nicolas MH, Nguyen J, Truffot C, Grosset J (1984 Jan 1). Survey of the pbenotypes of susceptibility to β-lactams in *Enterobacteriaceae* at the Pitie-Salpetriere Hospital.. J. Antimicrob Chemother..

[25] MacFaddin F.J. (2000). Biochemical tests for identification of medical bacteria..

[26] Ranjbar R., Karami A., Farshad S., Giammanco G.M., Mammina C. (2014). Typing methods used in the molecular epidemiology of microbial pathogens: a how-to guide.. New Microbiol..

[27] Ranjbar R., Naghoni A., Yousefi S., Ahmadi A., Jonaidi N., Panahi Y. (2013). The study of genetic relationship among third generation cephalosporin-resistant *Salmonella enterica* strains by ERIC-PCR.. Open Microbiol. J..

[28] Ranjbar R., Pezeshknejad P., Khamesipour F., Amini K., Kheiri R. (2017). Genomic fingerprints of *Escherichia coli* strains isolated from surface water in Alborz province, Iran.. BMC Res. Notes.

[29] Wayne PA Clinical and Laboratory Standards Institute: Performance standards for antimicrobial susceptibility testing. CLSI, 2010; 20th informational supplement. M100-S20.

[30] Sambrok J.F., Russel D.W. (2001). Molecular cloning: A laboratorymanual..

[31] Oliver A., Weigel L.M., Rasheed J.K., McGowan Jr J.E., Raney P., Tenover F.C. (2002). Mechanisms of decreased susceptibility to cefpodoxime in *Escherichia coli*.. Antimicrob. Agents Chemother..

[32] Fang H., Ataker F., Hedin G., Dornbusch K. (2008). Molecular epidemiology of extended-spectrum β-lactamases among *Escherichia coli* isolates collected in a Swedish hospital and its associated health care facilities from 2001 to 2006.. J. Clin. Microbiol..

[33] Kim J., Kang H.Y., Lee Y. (2008). The identification of CTX-M-14, TEM-52, and CMY-1 enzymes in *Escherichia coli* isolated from the Han River in Korea.. J. Microbiol..

[34] Jang J., Suh Y.S., Di D.Y., Unno T., Sadowsky M.J., Hur H.G. (2013). Pathogenic *Escherichia coli* strains producing extended-spectrum β-lactamases in the Yeongsan River basin of South Korea.. Environ. Sci. Technol..

[35] Dhanji H., Murphy N.M., Akhigbe C., Doumith M., Hope R., Livermore D.M., Woodford N. (2011). Isolation of fluoroquinolone-resistant O25b:H4-ST131 *Escherichia coli* with CTX-M-14 extended-spectrum β-lactamase from UK river water.. J. Antimicrob. Chemother..

[36] Toroglu S., Dincer S., Korkmaz H. (2005). Antibiotic resistance in gram negative bacteria isolated from Aksu river in (Kahramanmaras) Turkey.. Ann. Microbiol..

[37] Zurfluh K., Hächler H., Nüesch-Inderbinen M., Stephan R. (2013). Characteristics of extended-spectrum β-lactamase- and carbapenemase-producing *Enterobacteriaceae* isolates from rivers and lakes in Switzerland.. Appl. Environ. Microbiol..

[38] Tissera S., Lee S.M. (2013). Isolation of extended spectrum β-lactamase (ESBL) producing bacteria from urban surface waters in Malaysia.. Malays. J. Med. Sci..

[39] Muleta T., Debela E., Chali O.K. (2016). Antibiotic sensitivity pattern of pathogenic bacterial isolates of public health concern from Lake Hawassa water, Ethiopia.. Int J Water Res Environ Eng..

[40] Haque A., Yoshizumi A., Saga T., Ishii Y., Tateda K. (2014). ESBL-producing *Enterobacteriaceae* in environmental water in Dhaka, Bangladesh.. J. Infect. Chemother..

[41] Reinthaler F.F., Posch J., Feierl G., Wüst G., Haas D., Ruckenbauer G., Mascher F., Marth E. (2003). Antibiotic resistance of *E. coli* in sewage and sludge.. Water Res..

[42] Chatterjee R., Sinha S., Aggarwal S., Dimri A.G., Singh D., Goyal P., Abhishek M. (2012). Studies on susceptibility and resistance patterns of various *E. coli* isolated from different water samples against clinically significant antibiotics.. Int. J. Bioassays..

[43] Chen B., Zheng W., Yu Y., Huang W., Zheng S., Zhang Y., Guan X., Zhuang Y., Chen N., Topp E. (2011). Class 1 integrons, selected virulence genes, and antibiotic resistance in *Escherichia coli* isolates from the Minjiang River, Fujian Province, China.. Appl. Environ. Microbiol..

